# Risk factor analysis of disc and facet joint degeneration after intersegmental pedicle screw fixation for lumbar spondylolysis

**DOI:** 10.1186/s13018-022-03082-9

**Published:** 2022-04-22

**Authors:** Hao Meng, Yuan Gao, Peng Lu, Guang-Min Zhao, Zhi-Cheng Zhang, Tian-Sheng Sun, Fang Li

**Affiliations:** 1 Department of Orthopaedics, The 7th Medical Center of Chinese PLA General Hospital, No. 5 Nanmen Cang, Beijing, 100700 China; 2 Department of Gynecology and Obstetrics, The 1st Medical Center of Chinese PLA General Hospital, Beijing, China

**Keywords:** Spondylolysis, Pedicle screw fixation, Risk factors, Disc degeneration, Facet joint degeneration

## Abstract

**Background:**

Patients who do not respond to conservative treatment of the isthmus are often treated with surgery. We used direct repair plus intersegment pedicle screw fixation for the treatment of lumbar spondylolysis. The aim of this observational study was to assess the effects of this technique and evaluate various risk factors potentially predicting the probability of disc and facet joint degeneration after instrumentation.

**Methods:**

The study included 54 male L5 spondylolysis patients who underwent pars repair and intersegment fixation using pedicle screws. Bony union was evaluated using reconstruction images of computed tomography. Radiographic changes, including disc height, vertebral slip, facet joint and disc degeneration in the grade of adjacent and fixed segments, were determined from before to final follow-up. Logistic regression analysis was performed to identify factors associated with the incidence of disc and facet joint degeneration.

**Results:**

Bony union was achieved in all cases. Logistic regression analysis revealed that instrumentation durations of greater than 15.5 months and 21.0 months were significant risk factors for the incidence of L4/5 and L5S1 facet degeneration, respectively.

**Conclusions:**

Intersegmental pedicle screw fixation provides good surgical outcomes and good isthmic bony union rates in patients with lumbar spondylolysis. The duration of fixation was confirmed as a risk factor for facet joint degeneration. Once bony union is achieved, instrument removal should be recommended.

## Background

Lumbar spondylolysis refers to a defect of the vertebral pars interarticularis caused by stress fracture and occurs in approximately 6% of the general population, and the incidence of symptomatic spondylolysis is reported to be higher in athletes [1]. The lesion is mainly located at L5 (85–95%) and L4 (5–15%) but can occur at any level [2].

Generally, patients with low back pain due to spondylolysis are often managed conservatively with medication, physical therapy, and injection treatment [3, 4]. Surgery is indicated when in patients who experience no response to comprehensive conservative treatment for more than 6 months as well as those with persistent back pain and pars nonunion. Increasing pain, worsening of preexisting neurological impairment and progressive olisthesis are also indications for surgical treatment [3, 5].

In this study, we used direct repair plus intersegment pedicle screw fixation for the treatment of lumbar spondylolysis. This method fixes an active segment, which may limit the flexibility of the spine, increase the load on the adjacent segments of the fixed segment and cause adjacent segment degeneration (ASD) [6, 7]. However, pedicle screws can be removed when bony union is achieved, and whether it accelerates the degeneration of adjacent segments is unclear. To our knowledge, postoperative instability between adjacent segments, facet joint and disc degeneration have not been examined in a single-center study of cases in which lumbar intersegmental pedicle screw fixation was performed.

The purpose of this study was to reveal the effect of intersegmental pedicle screw fixation in the treatment of lumbar disc herniation and to evaluate various risk factors that may predict the possibility of degeneration of the intervertebral disc and facet joints.

## Materials and methods

### Patients

**Fig. 1:**
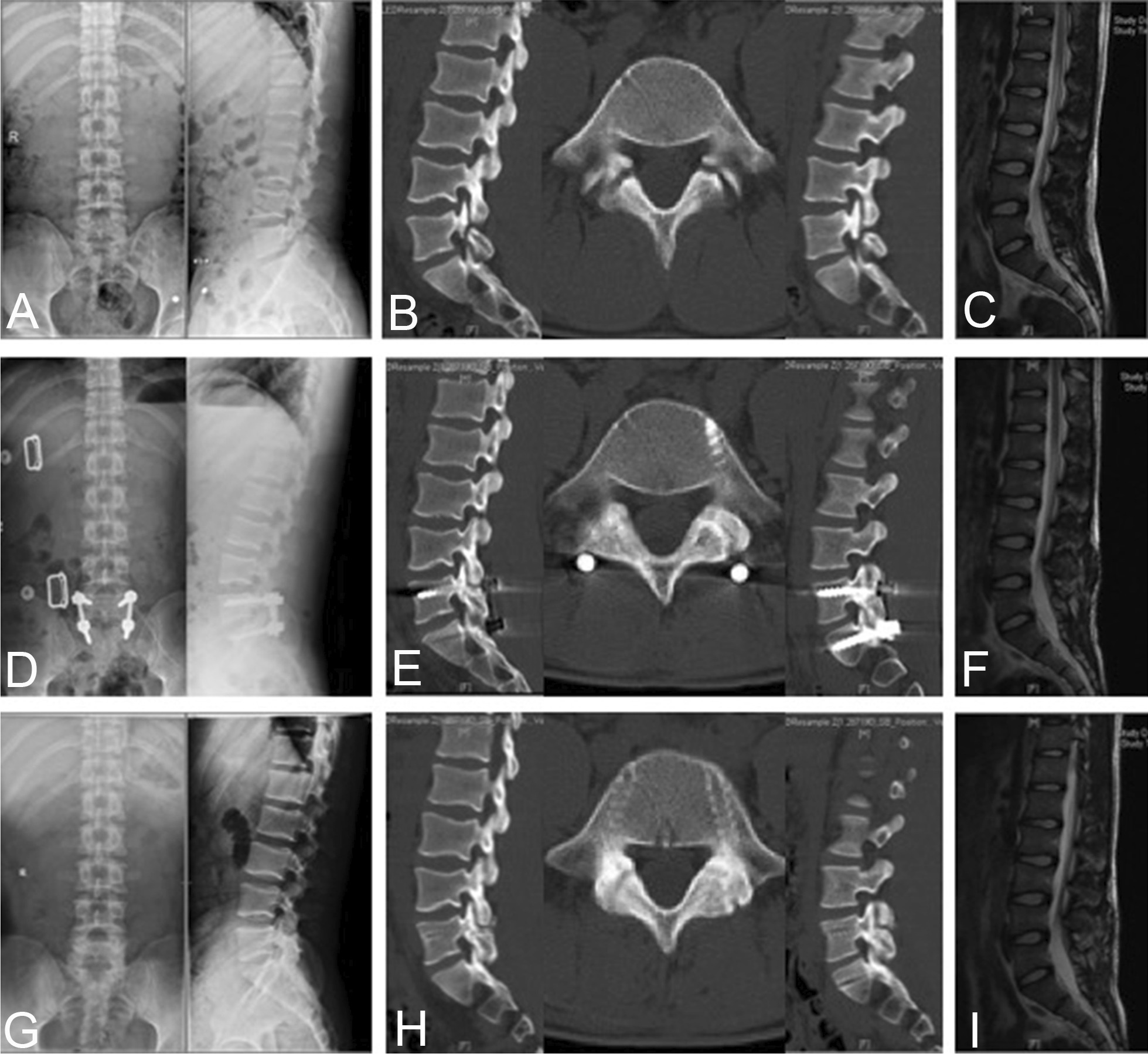
Preoperative and postoperative images obtained in a 21-year-old man with L5 spondylolysis. **a**–**c** Preoperative radiographs, CT scan and MRI. **d** One-week postoperative radiographs. **e** 12-Month follow-up CT scan showed bilateral bony union of the pars defect. **f** 15-Month postoperative MRI. **g** 15-Month postoperative radiographs showed that the lumbar instrumentations had been removed. **h**–**i** 15-Month postoperative CT scan and MRI

**Fig. 2:**
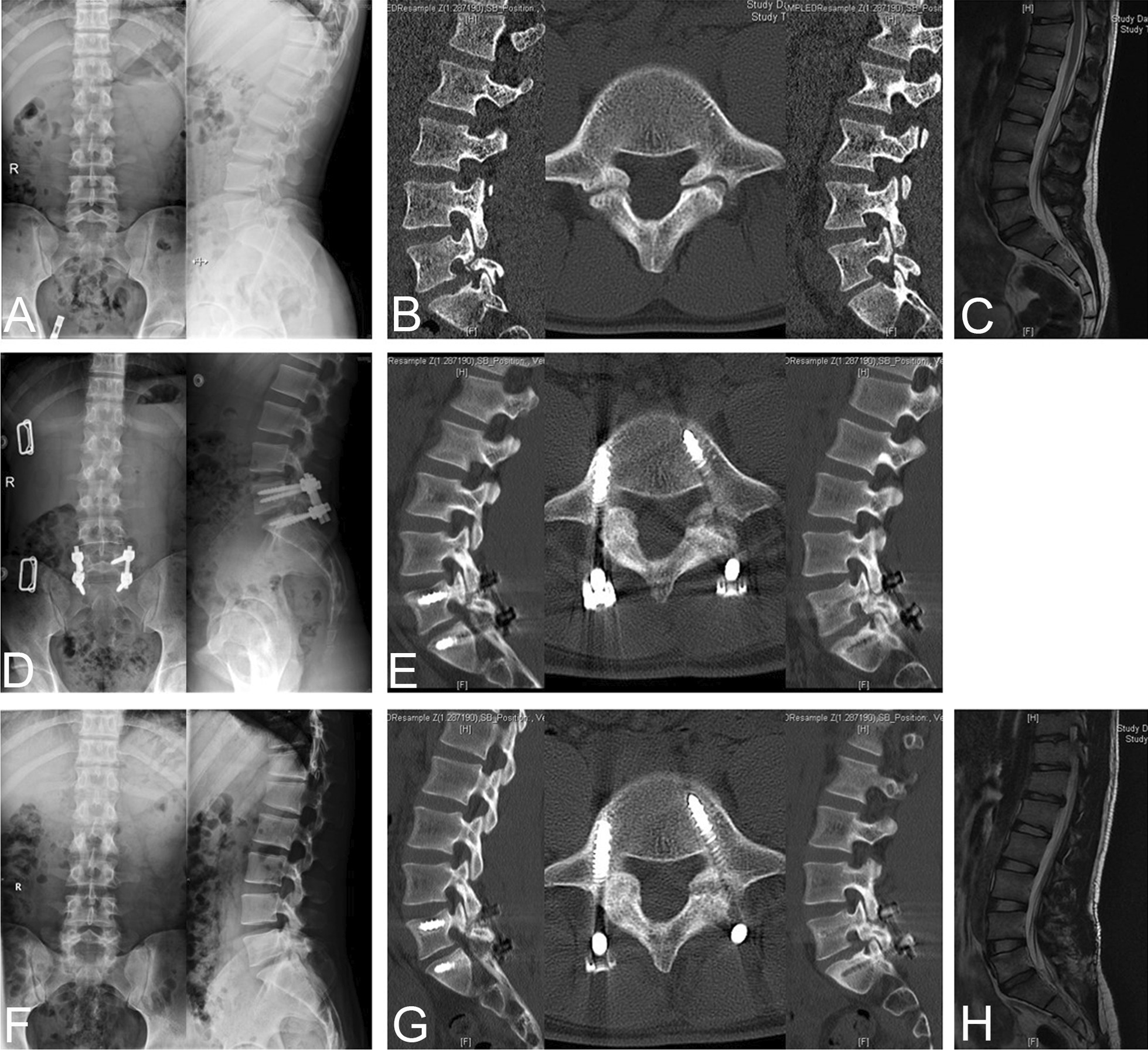
Preoperative and postoperative images obtained in a 19-year-old man with L5 spondylolysis. **a**–**c** Preoperative radiographs, CT scan and MRI. **d** One-week postoperative radiographs. **e** 9-Month follow-up CT scan. **f** 15-Month radiographs showed that the lumbar instrumentations had been removed. **g–h** 15-Month postoperative CT scan and MRI

We retrospectively evaluated consecutive spondylolysis patients who underwent pars repair and segmental fixation using pedicle screws at our institution between January 2016 and January 2018. All 54 patients were male, and the lesion was located at L5. Patients with symptomatic spondylolysis were treated with direct iliac bone graft repair combined with intersegmental pedicle screw fixation. Plain radiographs, computed tomography (CT) and magnetic resonance imaging (MRI) were evaluated at 6, 12, 18 and 24 months postoperatively. Instrumentation was removed after bony union was achieved (illustrative cases, Figs. 1 and 2). Local ethical committee and Institutional Research Board approval were obtained for the study.

Spondylolysis of the lumbar spine was diagnosed by five spine surgeons (HM, ZCZ, GMZ TSS and FL) as follows. All patients with low back pain had simple radiographs of the lumbar spine (anteroposterior, lateral and oblique views). If needed, a CT scan was performed to confirm the presence or absence of spondylolysis. In addition, patients with definite spondylosis on lumbar spine radiographs and CT scans underwent MRI of the lumbar spine to detect other spinal problems, such as disc degeneration and herniation. Surgical treatment was recommended for the following conditions: a patient with low back pain diagnosed with lumbar disc herniation, no response to conservative treatment at least 6 months, and MRI scans revealed no or mild degeneration of the intervertebral disc at the level of the pars defect. The exclusion criteria were radiological signs of spondylolisthesis or instability, previous spinal surgery, significant radicular pain, and improvement after conservative treatment.

### Operative procedures

The operation was performed under general anesthesia. The patient was placed in a prone position, and fluoroscopy was used to localize the surgical level. A 10-cm posterior midline incision was made. After incision of the lumbosacral fascia at 2 cm paraspinous process, the multifidus and longissimus muscles were identified and dissected away. The pars defects were exposed using curette and burr, carefully leaving facet joints intact. The lysis was prepared by removing fibrous tissue in and around the gap. The bony elements on both sides of the lysis were decorticated to assure bony healing of the lysis after pars repair. Cancellous bone grafts were harvested unilaterally from the iliac wing and implanted into the pars. Four pedicle screws were implanted to fix the isthmic vertebra and the adjacent distal vertebra. Instrumentation was removed after bony fusion was achieved.

### Radiological outcomes

Bony union was evaluated using CT reconstruction images at each follow-up. Reconstructions were made in both the axial and sagittal planes, allowing us to determine the degree of union.

From preoperative to final follow-up, imaging changes, such as disc height, vertebral slippage, facet joints and disc degeneration at adjacent and fixed levels, were determined. The relationship between CT and MRI findings and the progression of degeneration based on preoperative grading were also evaluated.

Disc height and vertebral slip at the L4/5 and L5/S1 levels were measured on lateral plain radiographs. The disc height was measured using Miyakoshi's procedures [8]. On the lateral radiograph, the following points to be marked were identified: the corners of the vertebral bodies, the midpoints of the endplates, and the midpoints of the walls of the vertebral bodies. The points were determined strictly according to the criteria of Quint et al. [9]. Using these easily defined points, the heights of the L4/5 and L5S1 discs were measured. The disc height (DH) was calculated as the mean of the anterior, middle and posterior disc heights. The sagittal diameter of the vertebral body from the anterior to posterior margin was measured at the mid-vertebral level, and the disc height index (DHI) was calculated as the disc height/sagittal diameter of the vertebral body [10].

Disc degeneration of the adjacent and fixed segments was reviewed using MRI during the follow-up. Disc degenerative grading was measured using the Pfirrmann 5-grade classification, which ranges from grades 1 to 5 [11]. Facet joint degeneration was measured using the classification for osteoarthritis of the Japanese Orthpaedic Association (JOA), which includes grades 0 to 4. Grade 0 indicates severe degeneration, whereas Grade 4 indicates a normal joint without degeneration [12].

All surgeries were performed using the same procedures by four spine surgeons (HM, ZCZ, GMZ and FL) at our institution, and the incidence of complications was compared between the two groups. Potential risk factors for disc and facet joint degeneration, such as age, obesity (body mass index, BMI), duration of fixation, vertebral slip and facet joint degeneration before primary surgery, were identified by reviewing medical records. Imaging assessment was assessed by two spine surgeons (ZCZ and GMZ). The inter-observer reliability was assessed by weighted kappa statistic. The degree of inter-observer reliability for qualitative measures was almost perfect for preoperative and end of follow-up measurements of facet degeneration grade on CT (pre: 0.795, end of follow-up: 0.854) and disc degeneration grade on MRI (pre: 0.821, end of follow-up: 0.833).

The demographic and clinical data of patients are in accordance with the normal distribution (Kolmogorov–Smirnov one-sample test, all *p* > 0.05). Student’s t test or analysis of variance was used for continuous variables, and Fisher’s exact test was used for categorical variables. SPSS software, version 23.0 (SPSS, Chicago, IL, USA), was used for all analyses, and a two-sided *p* value < 0.05 was considered statistically significant. Logistic regression analysis was performed to identify factors independently associated with the incidence of disc and facet joint degeneration.

## Results

At the time of the primary surgery, the mean age was 22.76 years (18 to 34). The mean BMI was 22.81 kg/m^2^ (19.23 to 24.77). Bony union was achieved in all cases as confirmed by CT reconstruction, and the mean fixed time was 17.04 months (8 to 39). No complications were observed during the follow-up.

Patient characteristics of disc and facet joint degeneration detected on plain radiographs are shown in Table 1. Decreased disc height in the upper adjacent segment L4/5 of ≥ 20% occurred in one patient, whereas decreased disc height in the fixed segment L5/S1 of ≥ 20% was observed in one patient. The disc height of the upper adjacent segment L4/5 was reduced by 20% to 10% in 7 patients, and 8 patients were observed in the fixed segment L5/S1. CT indicated facet joint degeneration at L4/5 and L5/S1 in 7 and 9 patients, respectively. MRI showed disc degeneration at L4/5 and L5/S1 in 5 patients, separately.

**Table 1:** Radiographic disc and facet joint degeneration

	L4/5 (%)	L5S1 (%)
*X-ray*		
DH: decrease ≥ 20%	1 (2%)	1 (2%)
DH: 20% ≥ decrease ≥ 10%	7 (13%)	8 (15%)
*CT*		
Facet degeneration increase one grade	7 (13%)	7 (13%)
Facet degeneration increase more than one grade	0	2 (4%)
*MRI*		
Disc degeneration increase one grade	5 (9%)	5 (9%)
Disc degeneration increase more than one grade	0	0

DH, disc height

The mean disc height in the upper adjacent segment L4/5 was 14.17 ± 1.74 before surgery and 14.01 ± 2.23 after removal of instruments. The mean DHI in L4/5 was 0.381 ± 0.03 before surgery and 0.370 ± 0.04 after removal of instruments. The mean disc height in the fixed segment L5/S1 was 14.13 ± 1.90 before surgery and 13.82 ± 2.03 after removal of instruments. The mean DHI in L5S1 was 0.389 ± 0.05 before surgery and 0.380 ± 0.05 after removal of internal fixation. No significant differences were observed (Table 2). There were 15 patients with a DHI decrease ≥ 10% and 39 patients with a DHI decrease ≤ 10%. Regarding the L5S1 level, the number of preoperative facet joint degeneration patients with a DHI reduction of ≥ 10% was 9 cases, and the number of patients with a DHI reduction of < 10% was 34 cases. The difference is significant (*p* < 0.05). The difference was significant (*p* < 0.05). Preoperative DHI decreased by ≥ 10% in 12 cases, and DHI decreased by less than 10% in 29 cases, but the difference was not significant (Table 3). Regarding the L4/5 level, no significant differences were observed between the DHI changes and preoperative facet joint and disc degeneration (Table 3).

**Table 2:** Radiographic changes of disc height on plain radiographs

	Pre-operation	Post-operation	*t* value	*p* value
DH (L4/5)	14.17 ± 1.74	14.01 ± 2.23	0.760	0.451
DHI (L4/5)	0.381 ± 0.03	0.370 ± 0.04	1.993	0.051
DH (L5S1)	14.13 ± 1.90	13.82 ± 2.03	1.712	0.093
DHI (L5S1)	0.389 ± 0.05	0.380 ± 0.05	1.772	0.082

DH, disc height; DHI, disc height index

**Table 3:** Relationship between preoperative disc and facet joint status and disc degeneration

	L4/5				L5S1			
	DHI decrease ≥ 10% (n)	DHI decrease < 10% (n)	*F *value	*p *value	DHI decrease ≥ 10% (n)	DHI decrease < 10% (n)	*F *value	*p* value
*Facet grade pre-operation*			0.000	1.000			4.934	0.026*
Degeneration	8	32			9	34		
No degeneration	3	11			6	5		
*Disc grade pre-operation*			0.002*	0.964			0.006	0.937
Degeneration	8	34			12	29		
No degeneration	3	9			3	10		

DH, disc height; DHI, disc height index

^*^Test was considered statistically significant at *p* < 0.05

In the analysis of L4/5 and L5S1 disc degeneration, no significant differences in demographic data (age, BMI, duration of instrument fixation, vertebral slip, and preoperative facet and disc degeneration change) were noted between the two groups (degeneration group and no degeneration group) (Table 4). In the analysis of L4/5 facet degeneration, no significant differences in demographic data (BMI, vertebral slip, and preoperative facet and disc degeneration change) were noted between the two groups (degeneration group and no degeneration group); however, the facet degeneration group showed a significantly older age and longer duration of instrument fixation than the no degeneration group (Table 5). In the analysis of L5S1 facet degeneration, no significant differences in demographic data (age, vertebral slip, and preoperative facet and disc degeneration change) were noted between the two groups (degeneration group and no degeneration group). The facet degeneration group showed a significantly higher BMI and longer duration of instrument fixation than the no degeneration group (Table 5).

**Table 4:** The analysis of risk factors for L4/5 and L5S1 discs

	L4/5				L5S1			
	Degeneration	No change		*p* value	Degeneration	No change		*p* value
Age (y)	25.60 ± 5.46	22.47 ± 4.42	1.479	0.145	23.00 ± 5.15	22.73 ± 4.55	0.123	0.903
BMI	22.34 ± 1.97	22.86 ± 0.91	− 1.070	0.290	22.69 ± 0.51	22.82 ± 1.08	− 0.257	0.799
Fixed time (m)	17.60 ± 1.97	16.98 ± 6.51	0.202	0.841	15.20 ± 4.97	17.29 ± 6.61	− 0.684	0.497
*Vertebral slip (n)*	5	49	0.117	0.732	5	49	0.000*	1.000
yes	1	19			2	18		
no	4	30			3	31		
*Facet grade pre-operation*	5	49	2.305	0.310			1.870	0.641
Grade 4	0	13			4	7		
Grade 3	5	28			7	14		
Grade 2	0	8			4	9		
Grade 1	0	0			1	8		
Grade 0	0	0			0	0		
*Disc grade pre-operation*	5	49	3.094	0.347	5	49	6.854	0.113
Grade 1	2	9			3	10		
Grade 2	2	26			0	22		
Grade 3	0	10			2	10		
Grade 4	1	4			0	6		
Grade 5	0	0			0	1		

BMI, Body Mass Index

^*^Test was considered statistically significant at *p* < 0.05

**Table 5:** The analysis of risk factors for L4/5 and L5S1 facet joints

	L4/5				L5S1			
	Degeneration	No change		*p* value	Degeneration	No change		*p* value
Age (y)	26.29 ± 5.38	22.23 ± 4.24	2.278	0.027*	23.22 ± 4.82	22.67 ± 4.56	0.331	0.742
BMI	23.37 ± 0.59	22.72 ± 1.06	1.572	0.122	23.49 ± 0.81	22.67 ± 1.03	2.225	0.030*
Fixed time (m)	25.86 ± 8.86	15.72 ± 4.98	4.495	0.000*	25.56 ± 4.67	15.33 ± 5.39	5.297	0.000*
*Vertebral slip (no.)*			0.000	1.000			1.922	0.166
Yes	3	17			1	19		
No	4	30			8	26		
*Facet grade pre-operation*			0.614	0.852			3.569	0.389
Grade 4	1	13			1	10		
Grade 3	5	27			4	17		
Grade 2	1	7			3	10		
Grade 1	0	0			1	8		
Grade 0	0	0			0	0		
*Disc grade pre-operation*			2.934	0.317			6.404	0.145
Grade 1	1	11			1	12		
Grade 2	2	26			6	16		
Grade 3	2	7			0	12		
Grade 4	2	7			2	4		
Grade 5	0	0			0	1		

BMI, Body Mass Index

^*^Test was considered statistically significant at *p* < 0.05

### Logistic regression analysis

**Fig. 3:**
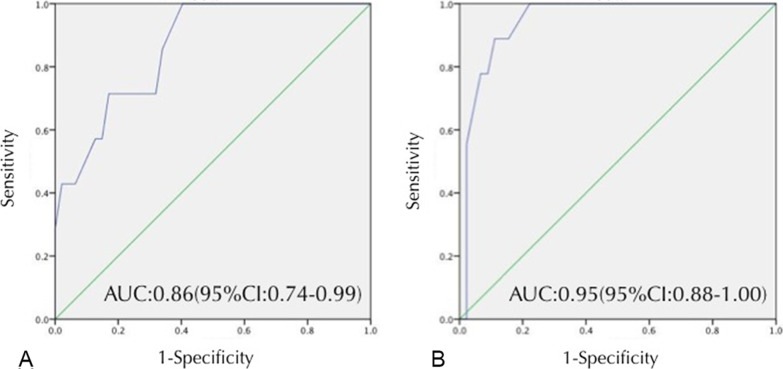
Logistic regression and ROC analysis. **a** L4/5: a cutoff value of 15.5 months at which classification according to fixed time yields a sensitivity of 100% and specificity of 60%. The area under the curve AUC is 0.80. **b** L5S1: a cutoff value of 21.0 months at which classification according to fixed time yields a sensitivity of 89% and specificity of 89%. The area under the curve AUC is 0.80

To reveal the relative impact of variables on facet and disc degeneration, logistic regression analysis was performed. The variables from the univariate analysis that were associated with the incidence of ASD included age, BMI, duration of instrument fixation, vertebral slip, and preoperative facet and disc degeneration change. Logistic regression analysis revealed that a duration of instrument fixation of greater than 15.5 months was a significant risk factor for the incidence of L4/5 facet degeneration (*p* = 0.006; odds ratio: 1.337, 95% CI 1.108–1.615). For the duration of fixation, a cutoff value of 15.5 months was determined to discriminate with the highest sensitivity, a receiver operating characteristic (ROC) curve specificity based on receiver operating characteristic (ROC) curve analysis. The area under the curve (AUC) was 0.86 (95% confidence interval 0.74–0.99). A fixation time longer than 21.0 months was a significant risk factor for L5S1 facet joint degeneration (*p* = 0.001; odds ratio: 1.379, 95% CI 1.133–1.679). The AUC was 0.95 (95% confidence interval 0.88–1.00) (Table 6 and Fig. 3).

**Table 6:** Multivariate logistic regression for facet joint degeneration

Level	Risk factor	*p* value	OR (95% CI)
L4/5	Fixed time	0.006	1.270 (1.072–1.503)
L5S1	Fixed time	0.001	1.379 (1.133–1.679)

Evaluation of aggravation based on preoperative grade suggested that patients with facet joint degeneration at L4/5 pre-operation exhibited no significant difference in greater progression of degeneration compared to patients without preoperative facet joint degeneration. The same results were noted for the L5S1 level. The preoperative grade of facet degeneration was not associated with the postoperative progression of disc degeneration, and preoperative disc degeneration exhibited no relationship with the postoperative progression of facet degeneration.

## Discussion

A considerable number of patients, especially young patients, complain of lumbar pain or have bilateral lumbar spondylolysis during physical examination, without or only mild lumbar spondylolisthesis, and usually have no neurological problems in imaging, symptoms and signs [13, 14]. However, consensus or guidelines for the treatment of these patients are not available. The treatment of simple bilateral lumbar spondylolysis can be divided into conservative treatment and surgical treatment [15]. The current clinically accepted view is that active surgical treatment should be adopted for patients who experience no improvement with conservative treatment for 6 months and are in the terminal stage of isthmic fissure on imaging [3, 16].

Previous studies have revealed a great number and variety of surgical techniques for spondylolysis repair, demonstrating the lack of consensus on a satisfactory procedure. Treatment mainly includes intersegmental fusion and intrasegmental isthmus repair [17]. For patients with simple lumbar spondylolisthesis, fusion has an effect on the fixation of the vertebral body, which can effectively prevent the diseased vertebral body from further spondylolisthesis. However, mobility of the fused segment is sacrificed, and the procedure may accelerate the degeneration of adjacent segments. Intrasegment repair is considered to preserve anatomical structure [18]. With the improvement in surgical techniques and the development of materials, surgical methods have gradually developed from early isthmic lag screws [19], transverse process–spinous process wires [20] and screw-hook constructs [21] to shaped rods and combination techniques. However, each of these approaches has limitations. The isthmus is too small to choose a suitable lag screw, and the lack of bone grafting in the isthmus leads to difficulties in bone healing. The wiring technique requires greater surgical exposure with extensive stripping of the muscle to expose the transverse process completely. The uneven force on the bilateral transverse processes can lead to complications, such as transverse process fractures and wire loosening, which may lead to nonunion of the pars defect. Gillet et al. [22] first used “V” rods to connect bilateral pedicle screws instead of laminar hook fixation. Ulibarri et al. [23] used a “U”-shaped titanium rod to connect bilateral pedicle universal screws and achieved a satisfactory fusion rate [24]. Although the hook-screw and shaped rod methods have achieved satisfactory results, a considerable number of spondylolysis patients are associated with laminosis or dysplasia of spinous processes [25], which may affect fixation strength. The use of minimally invasive techniques for the surgical management of spondylolysis has also been reported. Goldstein et al. used cortical screws and a spinous-process modular link in a minimally invasive fashion under intraoperative CT navigation [26]. Ghobrial et al. treated lumbar spondylolysis via a minimally invasive direct pars repair with cannulated screws [27].

In this study, compared with other techniques that require extensive muscle stripping, exposing the transverse process and injuring the interspinous ligament, intersegmental pedicle screws seem to be a technically simple and safe procedure. Easy surgical access through the Wiltse approach allows minimal soft tissue dissection and reduced blood loss. Hyperextension and rotation are the main stresses in the fatigue fracture of the isthmus. Compared with the above-mentioned various methods of intrasegment fixation, intersegment fixation provides stronger stability to resist rotation and extension. In addition, intersegment fixation compresses the isthmus, increases the degree of bone contact and promotes a higher fusion rate. Otherwise, for cases with spondylolisthesis within grade 1 and mild disc degeneration, motion segment fixation can correct and stabilize the spondylolisthesis. Compared with the method of intrasegmental fixation, it has the disadvantage of fixing the moving segment. Therefore, to restore the mobility of this segment, we removed the internal fixation early after CT confirmed complete isthmus healing.

Both facet joints and intervertebral discs are involved in the stability of the lumbar spine structure [28, 29] and are associated with degeneration of adjacent segments [30, 31]. To our knowledge, the incidence and risk factors for fixed and adjacent segment degeneration after intersegment pedicle screw fixation for lumbar spondylolysis have not been previously investigated. In this study, we identified the risk factors for facet joint and disc degeneration to predict and prevent this condition. After the follow-up of 54 patients, 100% bone healing was achieved. Only one patient exhibited a reduction in disc height of ≥ 20% in the upper and fixed segments. When the internal fixation was removed, we found a 9% incidence of grade 1 disc degeneration at the L4/5 and L5S1 levels (based on the Pfirrmann 5-grade classification system). Previous studies reported adjacent biomechanical alterations after lumbar fusion. Umehara et al. [32] reported that the load burden and weight shearing of the posterior column increased significantly at the adjacent segments. Weinhoffer et al. [33] also reported a significant increase in the disc pressure in the levels above the fused segments. In a systematic review, Harrop et al. [34] reported a 9% incidence of ASD after total disc replacement and a 34% incidence after fusion. We also found that 15% of the L4/5 level and 17% of the L5S1 level had a height reduction of greater than 10%, but the difference between pre- and postsurgery was not significant. The incidence of facet joint degeneration was 13% at the L4/5 level and 17% at the L5S1 level. Facet joint degeneration may arise from different mechanisms. First, surgical factors, such as damage to the articular process during screw placement, may lead to an increased risk of facet joint degeneration. Increased loading of the adjacent level after fixation also increases the load on the surrounding facet joints [35, 36]. Biomechanical studies have shown that the facet load can increase at the level of surgery after intervertebral fusion [37]. Although no intervertebral fusion was noted in this study, intersegmental fixation may also increase the load on the facet joint.

The ankylosis of facet joint articulation is a well-known consequence of spinal fusion procedures. Complete ankylosis of the zygapophyseal joints represents the realization of true spinal fusion [38]. In fact, the removal of the means of synthesis seems to slow down this process [39]. The results of this study suggest that fixation time is a risk factor associated with the development of facet degeneration. The nondegeneration cutoff value of fixed time has been hypothesized to be within 15.5 months. In fact, patients in this series with successful pars union presented a mean fixed time of 17.04 months. Therefore, regular postoperative follow-up is required to determine the bony union of the pars defect. Once bony union is achieved, removal of the internal fixation should be recommended. In addition, bone morphogenetic protein 2 (BMP-2) can be used to enhance fusion at an earlier time point [40]. The earlier bony union occurs, the lower the incidence of facet joint degeneration. Second, our results demonstrate that older age and higher BMI are associated with a higher incidence of facet joint degeneration at the L4/5 and L5S1 levels, respectively. In addition, sagittal balance and spinopelvic parameters also probably influence facet joint degeneration development [41]. Further study is needed to analyze spinopelvic parameters.

This study has some limitations. First, it was a retrospective study and was not performed as a comparative study. Second, the sample size in the present study was limited, which may increase the chances of making a type II error. Third, radiologic evaluations, including sagittal alignment assessment, were not performed.

## Conclusions

In this study, intersegmental pedicle screw fixation provided good surgical outcomes and good isthmic bony union rates in patients with lumbar spondylolysis. The duration of fixation was confirmed as a risk factor for facet joint degeneration. Once bony union is achieved, removal of the instruments is recommended.

## Abbreviations

ASD: Adjacent segment degeneration
CT: Computed tomography
MRI: Magnetic resonance imaging
DH: Disc height
DHI: Disc height index
JOA: Japanese Orthpaedic Association
BMI: Body Mass Index
AUC: Area under the curve
ROC: Receiver operating characteristic curve
BMP-2: Bone morphogenetic protein 2
